# Interdisciplinary Decision Making in Hemorrhagic Stroke Based on CT Imaging—Differences Between Neurologists and Neurosurgeons Regarding Estimation of Patients' Symptoms, Glasgow Coma Scale, and National Institutes of Health Stroke Scale

**DOI:** 10.3389/fneur.2019.00997

**Published:** 2019-09-26

**Authors:** Andrea Wagner, Karl-Michael Schebesch, Stefan Isenmann, Andreas Steinbrecher, Thomas Kapapa, Florian Zeman, Dobri Baldaranov, Oliver Grauer, Roland Backhaus, Ralf A. Linker, Felix Schlachetzki

**Affiliations:** ^1^Department of Neurology, University of Regensburg, Regensburg, Germany; ^2^Department of Neurosurgery, University Medical Center Regensburg, Regensburg, Germany; ^3^Department of Neurology, St. Josef Hospital, Moers, Germany; ^4^Department of Neurology, General Hospital Helios Klinikum Erfurt, Erfurt, Germany; ^5^Department of Neurosurgery, University Medical Center Ulm, Ulm, Germany; ^6^Center for Clinical Studies, University Medical Center Regensburg, Regensburg, Germany; ^7^Neurology, Department of Neurology and Institution for Translational Neurology, Münster, Germany; ^8^Stroke Center Hirslanden, Klinik Hirslanden Zurich, Zurich, Switzerland

**Keywords:** intracerebral hemorrhage, Glasgow coma scale, national institutes of health stroke scale, computed tomography, cerebral amyloid angiopathy, quality of life, outcome, telestroke

## Abstract

**Background and Purpose:** Acute intracerebral hemorrhage (ICH) requires rapid decision making toward neurosurgery or conservative neurological stroke unit treatment. In a previous study, we found overestimation of clinical symptoms when clinicians rely mainly on cerebral computed tomography (cCT) analysis. The current study investigates differences between neurologists and neurosurgeons estimating specific scores and clinical symptoms.

**Methods:** Overall, 14 neurologists and 15 neurosurgeons provided clinical estimates and National Institutes of Health Stroke Scale (NIHSS) as well as Glasgow Coma Scale (GCS) based on cCT images and basic information of 50 patients with hypertensive and lobar ICH. Subgroup analyses were performed for the different professions (neurologists vs. neurosurgeons) and bleeding subtypes (typical location vs. atypical). The differences between the actual GCS and NIHSS scores and the cCT-imaging-based estimated scores were depicted as Bland–Altman plots and negative and positive predictive value (NPV and PPV) for prediction of clinical relevant items. ΔNIHSS points (ΔGCS points) were calculated as the difference between actual and rated NIHSS (GCS) including 95% confidence interval (CI).

**Results:** Mean ΔGCS points for neurosurgeons was 1.16 (95% CI: −2.67–4.98); for neurologists, 0.99 (95% CI: −2.58–4.55), *p* = 0.308; mean ΔNIHSS points for neurosurgeons was −2.95 (95% CI: −12.71–6.82); for neurologists, −0.33 (95% CI: −9.60–8.94), *p* < 0.001. NPV and PPV for stroke symptoms were low, with large differences between different symptoms, bleeding subtypes, and professions. Both professions had more problems in proper rating of specific clinic–neurological symptoms than rating scores.

**Conclusion:** Our results stress the need for joint decision making based on detailed neurological examination and neuroimaging findings also in telemedicine.

## Introduction

Spontaneous intracerebral hemorrhages (ICHs) account for 10–15% of all strokes in Western populations, with a case fatality of 40−55% (1). Although treatment options are limited and neurosurgery is restricted to reducing mortality, rapid decision making is crucial for the individual patient. In stroke treatment, telemedical networks increasingly fill the void of specialized neurovascular centers and organize neurosurgical or neurological treatment. Cerebral computed tomography (cCT) images are primarily transferred to the specialist *via* telemedicine and ICH is easily identified as the cerebral pathology. Significant predictive outcome factors for ICH include the volume of blood on the initial cCT scan, presence, and ongoing expansion of intraventricular hemorrhage (IVH), hematoma location, and expansion and the neurological status (2). Further relevant predictors for outcome and functional independence after 100 days are the patient's age and the National Institutes of Health Stroke Scale (NIHSS) score at initial presentation, regardless of the location of the ICH (3, 4).

Often, patients are initially seen in primary care hospitals lacking specific neurological or neurosurgical departments. Non-specialized neurological examinations are increasingly followed by reading a patient's cCT imaging *via* teleradiologic consultation. In the case of ischemic stroke, the decision to conduct thrombolysis or even endovascular treatment basically relies on the NIHSS score, a cCT scan excluding ICH, an appropriate time window and the status of the arteries leading to the brain (5). In acute stroke therapy, telemedicine has been established in Germany in networks like the TeleMedical Project for integrative Stroke Care (TEMPiS) in Bavaria, Germany, for 15 years now and has significantly increased the rate of treated strokes and transient ischemic attacks as well as decreased the onset-to-treatment and door-to-needle time in clinically underserved areas (6). For clinical decision making, e.g., in thrombolysis, teleradiology using electronically transmitted original imaging data has to be completed by teleconsultation by a remotely located expert through the use of high-quality videoconferencing (7). The same concept could also be used for the first neurological consultation of patients with ICH in rural areas.

Especially in rural areas not having established telemedicine, often specialists in the tertiary centers are called by the local physicians and asked for their opinion without being able to do an examination of the patient by themselves—or patients have to be transferred to a center.

In a previous study we demonstrated that rating NIHSS and Glasgow Coma Scale (GCS) score merely according to the patients' cCT leads to underestimation of GCS and overestimation of NIHSS score, indicating that the patients' clinical symptoms were overestimated. This effect was particularly apparent in patients with lobar bleedings (8). The main objective of the present analysis was to evaluate possible differences between neurologists and neurosurgeons in estimation of patients' GCS and NIHSS score.

## Materials and Methods

Twenty board-certified neurologists and 20 board-certified neurosurgeons were given the anonymized files of the initial diagnostic cCT scans of 50 patients with an acute neurological deficit from ICH. No group of physicians was animated to do a special GCS or NIHSS training before participating in our study. Neurologists and neurosurgeons were blinded to the patients' specific clinical features but only given information about age of the patients and lapse of onset to scan, with the scan—apart from rare exceptions, e.g., in patients who came delayed and with an unusual anamnesis—being performed in the emergency department at latest 30 min after the patient's arrival, and were asked to estimate clinical symptoms (Table 1: questionnaire on the patients' symptoms to be filled in by the physicians) as well as the NIHSS and GCS score according to the cCT scan and the aforementioned basic clinical information. In the questionnaire are included basically the symptoms also found in the NIHSS score, but in a version also useable for the neurosurgeons not as used to NIHSS as neurologists. Therefore, we did not ask to graduate, e.g., hemiparesis like in the NIHSS score with 0–4 points but in absent–mild–severe—or even hemiplegia. We added items not included in the NIHSS score, but relevant to our everyday clinical treatment of ICH patients like anisocoria, reflexes, or the Babinski sign.

**Table 1:** Questionnaire on the patients' symptoms to be filled in by the physicians merely on the basis of the patients' basic characteristics (age, sex, time between symptom onset, and initial cCT scan) shown in Table 1 and the patients' initial cCT scans (English translated version).

Symptom	Yes	No
Reduced level of consciousness	1	1
Gaze palsy or forced deviation	1	1
Anisocoria	1	1
Mild impairment of orientation	1	1
Severe impairment of orientation	1	1
Aphasia/dysarthria	1	1
Neglect (extinction and inattention)	1	1
Mild paralysis	1	1
Partial paralysis	1	1
Complete paralysis	1	1
Sensory loss	1	1
Facial palsy	1	1
Hemianopia	1	1
Double vision	1	1
Limb ataxia	1	1
Stance and gait ataxia	1	1
Babinski's sign present	1	1
Elevated tendon reflexes	1	1
NIHSS	GCS	

The answers for the questionnaire, the physicians (neurologists and neurosurgeons) had to fill in, were sent back anonymized and we thus had no possibility to draw conclusions on any specified persons filling in these questionnaires.

According to the “(Model) Professional Code for Physicians in Germany–MBO-Ä 1997—The Resolutions of the 114th German Medical Assembly 2011 in Kiel as amended by the 118th German Medical Assembly 2015 in Frankfurt am Main, Art. 15-3 Research” performing research in Germany, you do not need an explicit vote of the ethic committee if you do not use person-specific data. For the same reason we decided not to obtain written consent of the participants. The participants were given oral and written information about the design and the aim of the study including anonymization.

Of the included patients, 25 suffered from an acute symptomatic deep, likely hypertensive, ICH in typical location as basal ganglia, pons, or cerebellum also classified as (deep) “typical” ICH due to the location (9). In an additional 25 patients, cCT scans depicted symptomatic lobar ICH in atypical location, therefore classified as “atypical” ICH. Patients suffering from ICH with other causes of ICH such as excessive administration of a vitamin K antagonist (defined as INR > 3), antecedent head trauma or ischemic stroke, CNS tumor, vascular malformation, vasculitis, blood dyscrasia, or coagulopathy were excluded (10). Patients with lobar ICH were only included if they had possible or probable cerebral amyloid angiopathy (CAA) according to the modified Boston criteria (11). These patients routinely underwent MRI (including FLAIR and T2^*^ susceptibility sequences) with MR-angiography or CT-angiography later on to rule out any vasculopathy and to detect microbleedings or lobar bleeds in cortical regions or cortical superficial siderosis, which defines probable CAA in the later course of the disease (11). Median age of the patients was 71 years (range: 39–97 years) and 26/50 were female. Time from symptom onset to initial cCT-imaging was <1.5 h in 12 patients, 1.5 to 4.5 h in 11 patients, 4.5 to 12 h in 11 patients, and more than 12 h in 16 patients (see Table 2). The patient data were only used in anonymized form to present them to the physicians, and the study did not affect individual patient treatment. Therefore, neither local ethic approval nor patient consent deemed necessary. All raters were informed about their participation in a study.

**Table 2:** Patient characteristics: age, type of ICH [a = atypical (highlighted in blue), t = typical (highlighted in red)], time between symptom onset and initial cCT scan, actual GCS/NIHSS score, medium GCS/NIHSS score estimated by the neurologists/neurosurgeons on the basis of the presented cCT scans and the basic information on the patient, ΔGCS/NIHSS score as the difference between actual and estimated GCS/NIHSS score.

**Pati-ent**	**Age (years)**	**Type of ICH**	**Time between symptom onset and initial cCT h**	**Actual NIHSS**	**Medium estimat-ed NIHSS**	**(Absolute) Δ NIHSS points**	**Actual GCS**	**Medium estimated GCS**	**(Absolute) Δ GCS points**
1	76–80	a	4.5–12	20	17.71	2.29	12	9.69	2.31
2	61–65	t	>12	13	16.60	3.60	12	9.83	2.17
3	81–85	a	4.5–12	6	5.30	0.70	15	13.52	1.48
4	66–70	a	>12	2	3.90	1.90	15	14.21	0.79
5	76–80	a	<1.5	10	3.20	6.80	12	13.93	1.93
6	61–65	t	>12	19	19.55	0.55	14	8.45	5.55
7	76–80	a	>12	3	4.00	1.00	15	14.10	0.90
8	71–75	t	<1.5	19	13.35	5.65	10	11.83	1.83
9	56–60	t	1.5–4.5	21	19.50	1.50	8	8.97	0.97
10	71–75	t	>12	4	7.95	3.95	15	13.96	1.04
11	41–45	t	>12	15	11.90	3.10	15	12.90	2.10
12	71–75	a	<1.5	12	7.16	4.84	15	12.85	2.15
13	86–90	t	>12	1	6.15	5.15	13	14.00	1.00
14	81–85	a	>12	7	11.45	4.45	11	12.07	1.07
15	81–85	a	4.5–12	4	6.60	2.60	14	13.62	0.38
16	61–65	a	< <1.5	4	4.80	0.80	15	13.97	1.03
17	56–60	a	<1.5	4	12.70	8.70	11	11.69	0.69
18	81–85	a	>12	24	14.10	9.90	11	11.52	0.52
19	66–70	a	4.5–12	13	13.30	0.30	15	11.14	3.86
20	91–95	t	1.5–4.5	12	11.68	0.32	10	12.41	2.41
21	71–75	a	1.5–4.5	3	3.20	0.20	15	14.52	0.48
22	96–100	a	1.5–4.5	20	25.90	5.90	7	5.52	1.48
23	91–95	t	1.5–4.5	7	15.45	8.45	14	10.90	3.10
24	66–70	a	1.5–4.5	16	20.85	4.85	10	7.45	2.55
25	86–90	t	1.5–4.5	16	18.05	2.05	15	10.79	4.21
26	81–85	a	<1.5	4	15.75	11.75	13	10.96	2.04
27	76–80	t	<1.5	22	22.53	0.53	8	7.78	0.22
28	66–70	a	<1.5	7	6.21	0.79	15	14.11	0.89
29	66–70	a	>12	3	5.70	2.70	15	13.96	1.04
30	61–65	a	4.5–12	3	7.35	4.35	15	13.82	1.18
31	71–75	a	1.5–4.5	11	13.00	2.00	15	11.54	3.46
32	51–55	t	<1.5	11	10.42	0.58	15	13.70	1.30
33	71–75	t	<1.5	23	22.10	0.90	8	9.07	1.07
34	76–80	a	4.5–12	4	8.80	4.80	13	13.68	0.68
35	36–40	t	1.5–4.5	25	22.55	2.45	8	7.75	0.25
36	81–85	t	4.5–12	8	11.15	3.15	15	13.43	1.57
37	66–70	t	<1.5	14	20.85	6.85	13	9.14	3.86
38	51–55	t	4.5–12	9	6.60	2.40	15	14.36	0.64
39	66–70	t	<1.5	13	16.45	3.45	15	9.86	5.14
40	86–90	t	4.5–12	27	20.45	6.55	7	7.64	0.64
41	61–65	t	4.5–12	11	11.20	0.20	14	13.29	0.71
42	71–75	t	>12	4	10.10	6.10	14	13.07	0.93
43	51–55	t	>12	3	8.25	5.25	15	13.68	1.32
44	71–75	a	>12	2	7.40	5.40	13	13.57	0.57
45	76–80	t	1.5–4.5	8	9.95	1.95	15	13.86	1.14
46	76–80	a	>12	15	8.00	7.00	14	12.70	1.30
47	56–60	t	>12	12	15.50	3.50	14	11.39	2.61
48	66–70	a	>12	3	3.70	0.70	15	14.39	0.61
49	61–65	a	4.5–12	2	11.65	9.65	15	11.21	3.79
50	51–55	t	1.5–4.5	22	13.10	8.90	10	12.64	2.64

The GCS and NIHSS scores rated by the physicians were compared to those calculated on the basis of the patients' medical records and clinical features reported in the patients' medical records at admission (here called “actual GCS/NIHSS”) analogous to Williams et al. (12). Mean ΔGCS points and mean ΔNIHSS points were defined as the difference between the actual GCS/NIHSS minus the rated GCS/NIHSS [ΔGCS points = GCS points (actual)—GCS points (rater); ΔNIHSS points = NIHSS points (actual) – NIHSS points (rater)].

Agreement between the actual and estimated NIHSS and GCS scores was evaluated on an individual patient basis, i.e., the patient's actual value was compared with one estimated value and calculated as the mean value of the estimate by all neurologists and neurosurgeons. The extent of agreement between the estimated and the actual NIHSS and GCS scores was quantified with the Bland–Altman plot (13). According to Krouwer (14), the actual NIHSS or GCS values rather than the average value were used on the *X*-axis. All plots include the mean difference, the 95% limits of confidence, and the regression line. All analyses were performed using R (version 3.3.3, The R Foundation for Statistical Computing).

In a next step, we analyzed whether or not neurological symptoms related to ICH existed that were easy to predict on the basis patients' cCT and basic clinical information only. Furthermore, we investigated whether a specific symptom can be predicted as present or absent.

Therefore, we calculated negative and positive predictive value (NPV and PPV) of the patients' symptoms on a single-item basis. PPV was defined as the probability that the patient in fact presented a symptom if the physician estimated the symptom to be present on the basis of the patient's cCT scan. NPV was defined as the probability that the physician predicted a symptom correctly as being absent. Furthermore, we performed subgroup analyses for neurologists vs. neurosurgeons and atypical vs. typical ICH using the same methods as described for the indicated subgroup. A PPV or NPV ≥ 0.7 was defined as acceptable by empirical reasons, a PPV or NPV > 0.8 as good, a PPV or NPV > 0.9 as very good.

## Results

Fourteen of 20 (70%) neurologists and 15 of 20 (75%) neurosurgeons addressed answered our questionnaire (see Table 3 for physician baseline data). The actual mean GCS score of the total ICH patient collective was 14 [standard deviation (*SD*) 2.5; range 7–15], and the actual mean NIHSS score was 10 (*SD* 7.3; range 1–27) (Table 2).

**Table 3:** Age (in age ranges of 5 years) and years of experience of board-certified neurologists and neurosurgeons—raw data.

**Physician (neur.,neurologist; n.surg. own, neurosurgeon from own hospital; n.surg. other, neurosurgeon from other hospital)**	**Group of physician (neur., neurologist; n.surg. own, neurosurgeon from own hospital; n.surg.other, neurosurgeon from other hospital)**	**Age**	**Years of practice after becoming board-certified neurologist/neurosurgeon**	**Years of leading a stroke unit (neurologists only)/number of operated ICH as board-certified neurosurgeon (neurosurgeons only)**
neur. 1	neur.	51–55	22	0
neur. 2	neur.	41–45	12	6
neur. 3	neur.	36–40	5	2
neur. 4	neur.	36–40	5	1
neur. 5	neur.	51–55	16	9
neur. 6	neur.	36–40	5	1
neur. 7	neur.	46–50	15	8
neur. 8	neur.	51–55	16	10
neur. 9	neur.	41–45	10	10
neur. 10	neur.	51–55	16	12
neur. 11	neur.	46–50	10	0
neur. 12	neur.	41–45	8	3
neur. 13	neur.	41–45	5	4
neur. 14	neur.	46–50	12	0
n.surg. own 1	n.surg. own	n.a.	n.a.	n.a.
n.surg. own 2	n.surg. own	n.a.	n.a.	n.a.
n.surg. own 3	n.surg. own	36–40	1	30
n.surg. own 4	n.surg. own	36–40	5	150
n.surg. own 5	n.surg. own	61–65	26	100
n.surg. own 6	n.surg. own	36–40	1.5	50
n.surg. own 7	n.surg. own	31–35	1	60
n.surg. own 8	n.surg. own	41–45	9	200
n.surg. own 9	n.surg. own	46–50	20	100
n.surg. own 10	n.surg. own	51–55	17	200
n.surg. other 1	n.surg. other	41–45	5	50
n.surg. other 2	n.surg. other	41–45	10	70
n.surg. other 3	n.surg. other	31–35	3	30
n.surg. other 4	n.surg. other	36–40	7	50
n.surg. other 5	n.surg. other	36–40	5	20

**Figure 1:**
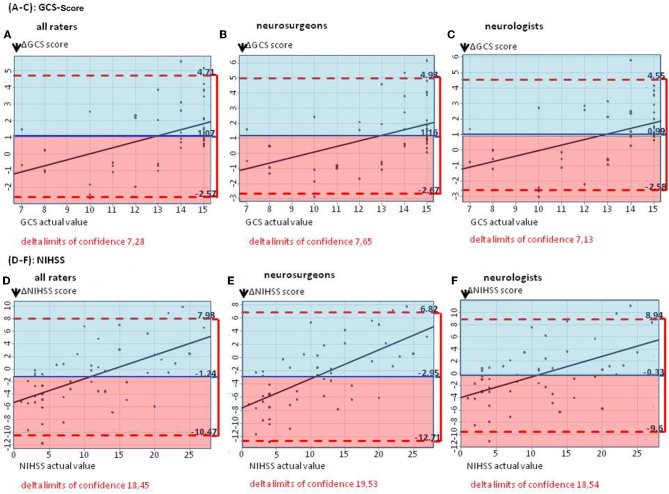
**(A–C)** The Bland–Altman plots show the difference between the actual and the estimated GCS score (mean for all raters) on patient basis: The mean estimated GCS score is too low by 1.07 GCS points **(A)**. **(B,C)** Subgroup analyses for estimated GCS score in neurosurgeons as well as neurologists, showing mean estimated scores too low by 1.16 and 0.99 GCS points, respectively. **(D–F)** The Bland–Altman plots show the difference between the actual and the estimated NIHSS score (mean for all raters) on patient basis: The median estimated NIHSS score is too high by 1.24 NIHSS points **(D)**. **(E,F)** Subgroup analyses. Neurologists overestimate NIHSS scores by 0.33 points, neurosurgeons by 2.95 points.

On average, GCS was rated too low by 1.07 points by all raters, with slightly lower results for neurosurgeons (mean ΔGCS points 1.16; 95% CI: −2.67, 4.98) than for neurologists (mean ΔGCS points: 0.99; 95% CI: −2.58, 4.55), *p* = 0.308 (Figures 1A–C). The NIHSS score estimated on the basis of the cCT correlated better with the actual values when rating was performed by neurologists (mean ΔNIHSS points: −0.33; 95% CI: −9.60, 8.94), than by neurosurgeons (mean ΔNIHSS points: −2.95; 95% CI: −12.71, 6.82; *p* < 0.001; for all raters: 1.24). Here, also the large CIs for all groups of physicians (all: 18.45; neurosurgeons: 19.53; neurologists: 18.54) reflected the challenge of properly estimating the patients' symptoms (Figures 1D–F).

Level of consciousness was the only item with a PPV > 0.7 in both groups of physicians (PPV = 0.74, Table 4). The lowest PPV observed were double vision, which the physicians failed to predict in >95% of cases (PPV 0.03) and anisocoria with a PPV of 0.10.

**Table 4:** NPV and PPV on a single-item basis (all types of ICH in all raters): Left column of the table showing the PPV with which the raters could predict a symptom, i.e., how likely it was that a patient in fact showed a symptom when the physician estimated the patient to have the symptom.

**PPV**		**NPV**	
Double vision	0.03	Hemihypesthesia	0.49
Anisocoria	0.10	Facial paresis	0.59
Ataxia of gait	0.31	Tendon reflexes reduced/elevated	0.64
Dysmetria	0.32	Reduced level of consciousness	0.65
Hemiplegia	0.33	Mild impairment of orientation	0.66
Mild impairment of orientation	0.35	Mild hemiparesis	0.69
Eye and head version	0.37	Severe hemiparesis	0.69
Severe hemiparesis	0.39	Babinski's sign present	0.71
Neglect	0.40	Neglect	0.72
Tendon reflexes reduced/elevated	0.42	Eye and head version	0.75
Mild hemiparesis	0.48	Dysmetria	0.75
Hemihypesthesia	0.48	Aphasia	0.77
Severe impairment of orientation	0.53	Severe impairment of orientation	0.79
Babinski's sign present	0.54	Scotoma	0.82
Scotoma	0.56	Ataxia of gait	0.83
Facial paresis	0.63	Hemiplegia	0.85
Aphasia	0.64	Anisocoria	0.90
Reduced level of consciousness	0.74	Double vision	0.97

*Right column of the table showing the NPV, i.e., how likely the symptom was absent when raters estimated it to be absent (highlighted in blue: NPV or PPV > 0.70, highlighted in red: NPV or PPV > 0.80, highlighted in green: NPV or PPV > 0.90)*.

The NPV, i.e., exclusion of symptoms based on cCT, were also relatively low with merely half of items correctly rated with a NPV of more than 0.7 and only 1/6 of items correctly rated with a NPV of more than 0.8 (Table 4). Only the symptoms double vision and anisocoria could be sufficiently excluded [NPV = 0.97 and 0.90, respectively (Table 4)]. One of the most important neurological symptoms—reduced level of consciousness—did not reach a NPV of >0.7 (NPV 0.65) (Table 4).

In order to decipher differences for typical vs. atypical ICH, we analyzed differences of PPV and NPV on a single-item basis (Tables 5A,B). The results were inhomogeneous with items reaching better results in scoring patients with either typical or atypical ICH. For the item aphasia as an example, a PPV of 0.76 for atypical ICH was reached (as opposed to <0.7 for typical ICH). In contrast, the item facial paresis had a PPV of 0.71 for typical ICH and < 0.7 for atypical ICH (Tables 5A,B).

**Table 5:** Subgroup analysis of PPV **(A)** and NPV **(B)** for atypical vs. typical ICH (data only shown for NPV or PPV > 0.70) (highlighted in blue: NPV or PPV > 0.70, highlighted in red: NPV or PPV > 0.80, highlighted in green: NPV or PPV > 0.90).

**Symptom**	**Typical ICH**	**Atypical ICH**	**PPV typical ICH > atypical ICH**	**PPV atypical ICH > typical ICH**
**(A) PPV** **>** **0.70**				
Facial paresis	0.71	<0.70	X	
Reduced level of consciousness	0.74	0.72	X	
Aphasia	<0.70	0.76		X
**Symptom**	**Typical ICH**	**Atypical ICH**	**NPV typical** **>** **atypical**	**NPV atypical** **>** **typical**
**(B) NPV** **>** **0.70**				
Reduced level of consciousness	<0.70	0.70		X
Eye and head version	0.72	0.81		X
Tendon reflexes reduced/elevated	<0.70	0.74		X
Neglect	<0.70	0.75		X
Hemiplegia	0.76	0.92		X
Mild hemiparesis	0.77	<0.70	X	
Dysmetria	<0.70	0.78		X
Severe impairment of orientation	0.79	0.78	X	
Scotoma	0.79	0.85		X
Aphasia	<0.70	0.82		X
Severe hemiparesis	<0.70	0.82		X
Babinski's sign present	<0.70	0.84		X
Anisocoria	0.85	0.96		X
Ataxia of gait	<0.70	0.88		X
Double vision	0.96	0.97		X

In general, NPV for neurological symptoms was better when rating atypical ICH than typical ICH for most items. Differences reached up to 15% between the two groups (Table 5B). The only two exceptions were mild hemiparesis (NPV for typical ICH 0.77, NPV for atypical ICH < 0.7) and severe impairment of orientation (NPV for typical ICH 0.79, NPV for atypical ICH 0.78), which reached a higher NPV in patients with typical ICH.

Level of consciousness in that subgroup analysis only reached a NPV of 0.70 for atypical ICH vs. < 0.70 in typical ICH (Table 5B).

Comparing neurologists and neurosurgeons, the PPV of neurologists were overall higher with four items >0.7. For neurosurgeons, only the item “level of consciousness” had a PPV > 0.7 (Table 6A). There was a slight trend for better rating of NPV by neurosurgeons than neurologists. Interestingly, rating the level of consciousness reached a NPV > 0.7 only in the group of neurologists [NPV 0.70 for neurologists, < 0.7 for neurosurgeons (Table 6B)].

**Table 6:** Subgroup analysis of PPV **(A)** and NPV **(B)** for neurologists vs. neurosurgeons (data only shown for NPV/PPV > 0.70) [highlighted in blue: NPV or PPV > 0.70, highlighted in red: NPV or PPV > 0.80, highlighted in green: NPV > 0.90 (for PPV: data >0.90 n.a.)].

**Symptom**	**Neurologist (neur.)**	**Neurosurgeon (n.surg.)**	**PPV neur. > n.surg**.	**PPV n.surg. > neur**.
**(A) PPV** **>** **0.70**				
Reduced level of consciousness	0.75	0.72	X	
Babinski's sign present	0.72	<0.70	X	
Facial paresis	0.81	<0.70	X	
Aphasia	0.83	<0.70	X	
**Symptom**	**Neurologist**	**Neurosurgeon**	**NPV neur**. **>** **n.surg**.	**NPV n.surg**. **>** **neur**.
**(B) NPV > 0.70**				
Reduced level of consciousness	0.70	<0.70	X	
Severe hemiparesis	<0.70	0.70		X
Dysmetria	0.78	0.72	X	
Eye and head version	0.76	0.73	X	
Facial paresis	<0.70	0.74	X	
Ataxia of gait	0.89	0.76	X	
Scotoma	0.78	0.86		X
Severe impairment of orientation	0.78	0.79		X
Neglect	<0.70	0.80		X
Tendon reflexes reduced/elevated	<0.70	0.80		X
Babinski's sign present	<0.70	0.82		X
Hemiplegia	0.88	0.83	X	
Anisocoria	0.89	0.91		X
Aphasia	<0.70	0.91		X
Double vision	0.96	0.98		X

Differences of more than 10% in rating NPV and PPV between neurologists and neurosurgeons are shown in Table 7.

**Table 7:** PPV (left column) and NPV (right column)—items with a difference of more than 10% in rating by neurologists vs. neurosurgeons.

**PPV**					**NPV**				
**Symptom**	**Neurologist**	**Neurosurgeon**	**PPV neur. > n.surg**	**PPV n.surg > neur**.	**Symptom**	**Neurologist**	**Neurosurgeon**	**NPV neur. > n.surg**	**NPV n.surg > neur**.
Severe impairment of orientation	0.58	0.48	X		Aphasia	0.65	0.91		X
Mild hemi paresis	0.57	0.39	X		Neglect	0.66	0.80		X
Facial paresis	0.81	0.42	X		Hemihyp estesia	0.31	0.69		X
Ataxia of gait	0.21	0.42		X	Facial paresis	0.45	0.74		X
Babinski's sign present	0.72	0.34	X		Ataxia of gait	0.89	0.76	X	
Tendon reflexes reduced/elevated	0.55	0.27	X		Babinski's sign present	0.60	0.82		X
					Tendon reflexes reduced/ elevated	0.50	0.80		X

## Discussion

In this study, we demonstrate distinct differences in estimating GCS and NIHSS scores based on cCT scan analysis among neurologists and neurosurgeons in ICH patients. In general, physicians had more problems in proper rating of specific clinical–neurological symptoms than of GCS and NIHSS scores. This scenario is often present in telemedical and even more pure teleradiological networks and may lead to unnecessary or erroneous transfer of patients stressing the need for joint decision making on the basis of thorough neurological investigation and communication. Even in hemorrhagic stroke, which is readily depicted on cCT, clinical neurological assessment cannot be replaced, as previously demonstrated (8).

The only clinical item that could be predicted with a PPV > 0.7, i.e., 70% correct prediction, was the level of consciousness, which is critical for intubation and thus of particular relevance, including outcome (2, 15–17). In the subgroup of atypical ICH, aphasia could be predicted with a PPV of 0.76. This is probably due to the relatively well-defined cortical presentation of the language-relevant structures resulting in aphasia. Especially in smaller ICH, eloquent areas are damaged more frequently by atypical than by typical ICH. In the subgroup of typical ICH, facial paresis reached a PPV of 0.71. Interestingly, this contrasts a former study showing that indeed facial paresis is quite hard to detect correctly even in clinical examination (18). A possible explanation is that brachiofacial hemiparesis is quite often due to damage of the white matter, the main location of typical ICH not only in our study, making a correct estimation of this symptom more likely.

The excellent NPV of 0.97 for double vision (in 97% of patients double vision was correctly excluded) may also result from the well-defined anatomical location of corresponding lesions in the brainstem or pons. Similarly, lesions causing anisocoria, also associated with a NPV of 0.9, typically involve brainstem or diencephalic lesions. This is of importance as unilateral dilation in an ICH patient is indicative for temporal herniation, is accompanied with a low GCS, and calls for immediate treatment [intubation, treatment in a (neuro-)intensive department, immediate CT control, decompressive surgery] (19–21).

Of the 11 of total 18 symptoms with a NPV > 0.7, 10 symptoms were associated with a PPV <0.6 (see Table 8). For 12 symptoms, PPV was even below 0.5, which would be expected for mere guessing. This indicates for a possible bias toward overestimation of symptoms by the rating physicians. The NPV was higher than PPV for 16 of the 18 symptoms. This also is in agreement with our former study, where we already showed that in rating GCS and NIHSS scores, physicians overestimated the patients' symptoms (8).

**Table 8:** Comparison of PPV and NPV on a single-item level [highlighted in blue: NPV or PPV > 0.70, highlighted in red: NPV > 0.80 (PPV n.a.), highlighted in green: NPV > 0.90 (PPV n.a.)].

**Symptom**	**PPV**	**NPV**	**NPV > PPV**	**NPV < PPV**	**NPV > 70**	**PPV > 60**
Double vision	0.03	0.97	X		X	
Anisocoria	0.10	0.90	X		X	
Ataxia of gait	0.31	0.83	X		X	
Dysmetria	0.32	0.75	X		X	
Hemiplegia	0.33	0.85	X		X	
Mild impairment of orientation	0.35	0.66	X			
Eye and head version	0.37	0.75	X		X	
Severe hemiparesis	0.39	0.69	X			
Neglect	0.40	0.72	X		X	
Tendon reflexes reduced/elevated	0.42	0.64	X			
Mild hemiparesis	0.48	0.69	X			
Hemihypesthesia	0.48	0.49	X			
Severe impairment of orientation	0.53	0.79	X		X	
Babinski's sign present	0.54	0.71	X		X	
Scotoma	0.56	0.82	X		X	
Facial paresis	0.63	0.59		X		X
Aphasia	0.64	0.77	X		X	X
Reduced level of consciousness	0.74	0.65		X		X

Overestimation of symptoms may lead to more inappropriate transfers of patients to tertiary centers or to neurosurgical departments posing medical, economical, and ethical problems. Any transfer of a severely ill patient carries an inherent medical risk and should be avoided and likewise surgical treatment should be based on relevant information to be outweighted with the operative risks. A recently published, retrospective study examined which factors are associated with emergent intervention during the first 24 h following helicopter transport of ICH patients to a tertiary-level care center. Age, GCS, and clot volume were detected as significant predictors of neurosurgical intervention within 24 h after helicopter transport, whereas in multivariate analysis, only younger age, GCS of 3–8, and lobar hemorrhage were found to be independent predictors for surgery. Interestingly, only 30.8% of the transferred patients had at least one neurosurgical intervention (22). Another study indicated that patients undergoing surgery have—even with similar ICHs—a longer stay on ICU (intensive care unit) and on mechanical ventilation (23). Patient transfers and surgery not indicated are also associated with high costs, which would be better invested in other medical treatments, e.g., for ensuing rehabilitation in these patients.

On the other hand, besides the initial cCT, other criteria like patient age and comorbidities are essential for the patient's prognosis, and in certain constellations, these criteria may lead to transfer even of a patient with only a minor ICH in initial cCT but a high risk for future deterioration due to secondary progress of ICH or edema.

Neurologists and neurosurgeons use different scoring systems in their everyday practice (8). Thus, the larger problems neurosurgeons had in rating NIHSS score compared to neurologists may be explained by the fact that both professions are well-acquainted with GCS score, whereas NIHSS score is typically used by clinical neurologists in everyday practice in patients with an ischemic stroke, where the NIHSS is known as a well-established score, also for the patients' future prognosis. Due to everyday practice, neurologists and neurosurgeons may have different points of view on the same patient: For the neurosurgeon, the most important question in emergency situations is whether or not the patient should undergo surgery. This is often decided depending on critical clinical signs such as altered level of consciousness or anisocoria, indicating live-threatening ICH. The other items may be more relevant to the neurologist, who in his everyday practice is more interested in the pathophysiologic background of the ICH as basis for adequate secondary prevention and long-term outcome and therefore has to be more aware also of minor deficits (24–27).

Several limitations of this study exist: first, the patient's GCS encompassed a range of 7–15 points, thus not representing the entire range of clinical presentations, and not reflecting the worst-case scenario in ICH. However, our spectrum of ICH cases represents the group of patients with the most uncertain prognosis, which may especially benefit from balanced decision making. Patients with a GCS of 8 and lower are often intubated already in the prehospital stage and have the highest probability of neurosurgery. But both in the surgery and conservative group, they have a very bad prognosis. Therefore, immediate palliative care is sometimes decided in these patients, too (5, 27, 28).

In the two largest randomized trials comparing early surgery (within 24 h of randomization) with medical treatment for spontaneous supratentorial intracerebral hematomas (STICH) and for spontaneous supratentorial lobar intracerebral hematomas (STICH II), patients also were only included with a GCS ≥ 5 (for STICH) and ≥7 (for STICH II), respectively (27, 28). Patients in our study had a higher overall GCS than in the STICH studies. For those, the inclusion criterion was “the clinical uncertainty principle,” i.e., if the responsible neurosurgeon was uncertain about the benefits of either treatment, the patient was included in the study. STICH did not show an overall benefit of early surgery, except for the subgroup of patients with hematoma within 1 cm of cortical surface. STICH II subsequently showed that early surgery might indeed have a small but clinically relevant survival advantage for patients with lobar ICH. The need for a rescue operation in the initial conservative treatment group and outcome in both groups were significantly correlated with initial clinical deficits of the patients. Both these and our study underline the importance of a good clinical examination of the patients prior to a decision on surgery (27, 28). The results are not strong enough to draw conclusions for guidelines. Present expert consensus is to consider surgery in superficial lobar clots, especially those larger than 30 ml and as a life-saving treatment in patients with GCS < 8 (5, 29).

Due to the small sample size, the statistical power of our study is limited. However, each participant examined cCT scans of 50 patients with completely more than 1,000 images for this study. As the acute decisions on patient care are often up to neurologists/neurosurgeons without 24/7 neuroradiological competence available, we decided against participation of neuroradiologists.

Furthermore, for the atypical ICH, only ICHs due to CAA were included. Our attempt was to create a more homogeneous collective of atypical ICH, better comparable to the etiologically quite homogenous collective of typical ICH. All the same, we cannot exclude that the generalizability of the study is limited by that.

As a further limitation, it can be discussed that the physicians did not have to participate in a systematic NIHSS training.

Our study underlines the necessity for a thorough neurological status in addition to the cCT scans despite the acute nature of the disease, even more in patients with atypical ICH, and the importance of multidisciplinary approach of neurologists, neurosurgeons, and physicians experienced in (neuro)radiology for best decision making.

## Data Availability Statement

The raw data supporting the conclusions of this manuscript will be made available by the authors, without undue reservation, to any qualified researcher.

## Author Contributions

AW, K-MS, and FS contributed conception and design of the study, and analysis and interpretation of the data. AW and FS wrote the first draft of the manuscript. FZ contributed analysis and interpretation of the data and wrote parts of the manuscript. SI, AS, TK, DB, OG, RB, and RL wrote sections of the manuscript. All authors contributed to manuscript revision, read, and approved the submitted version.

### Conflict of Interest

The authors declare that the research was conducted in the absence of any commercial or financial relationships that could be construed as a potential conflict of interest.

## References

[1] Samaraskera N Fonville A Lerpiniere C Farrall AJ Wardlaw JM White PM RAS for the lothian audit of the treatment of cerebral hemorrhage collaborators, influence of intracerebral hemorrhage location on incidence, characteristics, and outcome: population-based study. Stroke. (2015) 46:361–8. 10.1161/STROKEAHA.114.007953 25586833

[2] Hemphill J Bonovich DC Besmertis L Manley GT Johnston C. The ICH score - a simple, reliable grading scale for intracerebral hemorrhage. Stroke. (2001) 32:891–7. 10.1161/01.STR.32.4.891 11283388

[3] Shaya M Dubey A Berk C Gonzalez-Toledo E Zhang J Caldito G. Factors influencing outcome in intracerebral hematoma: a simple, reliable, and accurate method to grade intracerebral hemorrhage. Surg Neurol. (2005) 63:343–8. 10.1016/j.surneu.2004.06.019 15808717

[4] Weimar C Roth M Willig V Kostopoulos P Benemann J Diener HC. Development and validation of a prognostic model to predict recovery following intracerebral hemorrhage. J Neurol. (2006) 253:788–93. 10.1007/s00415-006-0119-x 16525882

[5] Powers WJ Derdeyn CP Biller J Coffey CS Hoh BL Jauch EC. 2015 American Heart Association/American Stroke Association focused update of the 2013 guidelines for the early management of patients with acute ischemic stroke regarding endovascular treatment. A guideline for healthcare professionals from the American Heart Association/American Stroke Association. Stroke. (2015) 46:3020–35. 10.1161/STR.0000000000000074 26123479

[6] Müller-Barna P Huber G Boy S Bogdahn U Wiedmann S Heuschmann PU. TeleStroke units serving as a model of care in rural areas-−10-year experience of the TeleMedical project for integrative stroke care. Stroke. (2014) 45:2739–44. 10.1161/STROKEAHA.114.006141 25147327

[7] Audebert HJ Schwann L. Telestroke: scientific results. Cerebrovasc Dis. (2009) 27 (Suppl. 4):15–20. 10.1159/000213054 19546537

[8] Wagner A Schebesch KM Zeman F Isenmann S Steinbrecher A Kapapa T. Primary cCT imaging based clinico-neurological assessment—Calling for addition of telestroke video consultation in patients with intracerebral hemorrhage. Front Neurol. (2018) 9:607. 10.3389/fneur.2018.00607 30093878 PMC6071543

[9] Godoy D Pinero GR Koller P Masotti L Di Napoli M. Steps to consider in the approach and management of critically ill patient with spontaneous intracerebral hemorrhage. World J Crit Care Med. (2015) 4:213–29. 10.5492/wjccm.v4.i3.213 26261773 PMC4524818

[10] Martí-Fàbregas J Prats-Sánchez L Martínez-Domeno A Camps-Renom P Marín R Jiménez-Xarrié E. The H-ATOMIC criteria for the etiologic classification of patients with intracerebral hemorrhage. PLoS ONE. (2016) 11:e0156992. 10.1371/journal.pone.0156992 27275863 PMC4898692

[11] Charidimou A Gang Q Werring DJ. Review—Sporadic cerebral amyloid angiopathy revisited: recent insights into pathophysiology and clinical spectrum. J Neurol Neurosurg Psychiatry. (2012) 83:124–37. 10.1136/jnnp-2011-301308 22056963

[12] Williams L Yilmaz EY Loez-Yunez AM. Retrospective assessment of initial stroke severity with the NIH stroke scale. Stroke. (2000) 31:858–62. 10.1161/01.STR.31.4.858 10753988

[13] Bland J Altman DG. Statistical methods for assessing agreement between two methods of clinical measurement. Lancet. (1986) 1:307–10. 10.1016/S0140-6736(86)90837-8 2868172

[14] Krouwer J. Why Bland–Altman plots should use X, not. (Y+X)/2 when X is a reference method. Stat Med. (2008) 27:778–80. 10.1002/sim.3086 17907247

[15] Hemphill JF Neill TA Prospective validation of the ICH Score for 12-months functional outcome. Neurology. (2009) 73:1088–94. 10.1212/WNL.0b013e3181b8b332 19726752 PMC2764394

[16] Hoffmann M Czorlich P Lehmann W Spiro AS Rueger JM Lefering R The impact of prehospital intubation with and without sedation on outcome in trauma patients with a GCS of 8 or less. J Neurosurg Anesthesiol. (2017) 29:161–7. 10.1097/ANA.0000000000000275 26797107

[17] Ruiz-Sandoval J Chiquete E Romero-Vargas S Padilla-Martínez JJ Gonzáles-Cornejo S. Grading scale for prediction of outcome in primary intracerebral hemorrhages. Stroke. (2007) 38:1641–4. 10.1161/STROKEAHA.106.478222 17379820

[18] Josephson SA Hills NK Johnston SC. NIH stroke scale reliability in ratings from a large sample of clinicians. Cerebrovasc Dis. (2006) 22:389–95. 10.1159/000094857 16888381

[19] Kilincer C Asil T Utku U Hamamcioglu MK Turgut N Hicdonmez T. Factors affecting the outcome of decompressive craniectomy for large hemispheric infarctions: a prospective cohort study. Acta Neurochir. (2005) 147:587–94. 10.1007/s00701-005-0493-7 15739038

[20] Mojumder DK Patel S Nugent K Detoledo J Kim J Dar N. Pupil to limbus ratio: introducing a simple objective measure using two-box method for measuring early anisocoria and progress of pupillary change in the ICU. J Neurosci Rural Pract. (2015) 6:208–15. 10.4103/0976-3147.153229 25883482 PMC4387813

[21] Vedantam A Robertson CS Gopinath SP. Clinical characteristics and temporal profile of recovery in patients with favorable outcomes at 6 months after severe traumatic brain injury. J Neurosurg. (2018) 129:234–40. 10.3171/2017.3.JNS162720 28937323

[22] D'Agostino E Hong J Sudoko C Simmons N Lollis SS. Prehospital predictors of emergent intervention after helicopter transfer for spontaneous intraparenchymal hemorrhage. World Neurosurg. (2018) 120:e274–81. 10.1016/j.wneu.2018.08.050 30142435

[23] Stein M Misselwitz B Hamann GF Kolodziej MA Reinges MH Uhl E. Defining prolonged length of acute care stay for surgically and conservatively treated patients with spontaneous intracerebral hemorrhage: a population-based analysis. Biomed Res Int. (2016) 2016:9095263. 10.1155/2016/9095263 27110572 PMC4826712

[24] Cho DY Chen CC Lee HC Lee WY Lin HL. Glasgow Coma Scale and hematoma volume as criteria for treatment of putaminal and thalamic intracerebral hemorrhage. Surg Neurol. (2008) 70:628–33. 10.1016/j.surneu.2007.08.006 18207500

[25] Gautschi OP Schaller K. Surgery or conservative therapy for cerebral haemorrhage? Lancet Neurol. (2013) 382:P377–8. 10.1016/S0140-6736(13)61087-9 23726394

[26] Hemphill JC Greenberg SM Anderson CS Becker K Bendok BR Cushman M. Guidelines for the management of spontaneous intracerebral hemorrhage: a guideline for healthcare professionals from the American Heart Association/American Stroke Association. Stroke. (2015) 46:2032–60. 10.1161/STR.0000000000000069 26022637

[27] Mendelow AD Gregson BA Fernandes HM Murray GD Teasdale GM Hope DT. Early surgery versus initial conservative treatment in patients with spontaneous supratentorial intracerebral haematomas in the International Surgical Trial in Intracerebral Haemorrhage (STICH): a randomised trial. Lancet Neurol. (2005) 365. 10.1016/S0140-6736(05)17826-X 15680453

[28] Mendelow AD Gregson BA Rowan EN Murray GD Gholkar A Mitchell PM. Early surgery versus initial conservative treatment in patients with spontaneous supratentorial lobar intracerebral haematomas (STICH II): a randomised trial. Lancet Neurol. (2013) 382:387–97. 10.1016/S0140-6736(13)60986-1 23726393 PMC3906609

[29] Trabert J Steiner T. Medical versus surgical management of intracerebral hematomas. Curr Atheroscler Rep. (2012) 14:366–72. 10.1007/s11883-012-0259-7 22700472

